# Testing Causal Effects of Maternal Smoking During Pregnancy on Offspring’s Externalizing and Internalizing Behavior

**DOI:** 10.1007/s10519-015-9738-2

**Published:** 2015-09-01

**Authors:** C. V. Dolan, L. Geels, J. M. Vink, C. E. M. van Beijsterveldt, M. C. Neale, M. Bartels, Dorret I. Boomsma

**Affiliations:** Department of Biological Psychology, Netherlands Twin Register, VU University Amsterdam, Van der Boechorststraat 1, 1081 BT Amsterdam, The Netherlands; Virginia Institute for Psychiatric and Behavioral Genetics, Virginia Commonwealth University, Richmond, VA USA; EMGO+ Institute for Health and Care Research, VU University Medical Centre, Amsterdam, The Netherlands; Neuroscience Campus Amsterdam, Amsterdam, The Netherlands

**Keywords:** Parental prenatal smoking, Pleiotropic effects, Childhood behavioral problems, Causality

## Abstract

Maternal smoking during pregnancy (SDP) is associated with increased risk of externalizing and internalizing behaviors in offspring. Two explanations (not mutually exclusive) for this association are direct causal effects of maternal SDP and the effects of genetic and environmental factors common to parents and offspring which increase smoking as well as problem behaviors. Here, we examined the associations between parental SDP and mother rated offspring externalizing and internalizing behaviors (rated by the Child Behavior Checklist/2–3) at age three in a population-based sample of Dutch twins (N = 15,228 pairs). First, as a greater effect of maternal than of paternal SDP is consistent with a causal effect of maternal SDP, we compared the effects of maternal and paternal SDP. Second, as a beneficial effect of quitting smoking before pregnancy is consistent with the causal effect, we compared the effects of SDP in mothers who quit smoking before pregnancy, and mothers who continued to smoke during pregnancy. All mothers were established smokers before their pregnancy. The results indicated a greater effect of maternal SDP, compared to paternal SDP, for externalizing, aggression, overactive and withdrawn behavior. Quitting smoking was associated with less externalizing, overactive behavior, aggression, and oppositional behavior, but had no effect on internalizing, anxious depression, or withdrawn behavior. We conclude that these results are consistent with a causal, but small, effect of smoking on externalizing problems at age 3. The results do not support a causal effect of maternal SDP on internalizing behaviors.

## Introduction

The aim of the present paper is to test two hypotheses concerning the relationship between maternal smoking during pregnancy (SDP) and offspring internalizing and externalizing behavioral problems in a Dutch sample of 3 year old twins and their parents. While the association between maternal SDP and offspring externalizing behaviors is well established (Gaysina et al. 2013; Thapar et al. 2009; D’Onofrio et al. 2008; Langley et al. 2012; Nomura et al. 2010; Keyes et al. 2014; Tiesler et al. 2011; Linnet et al. 2003; Cornelius et al. 2011; Lavigne et al. 2011; Paradis et al. 2011; Gatzke-Kopp and Beauchaine 2007; Brion et al. 2010; Stene-Larsen et al. 2009; for a review, see Tiesler and Heinrich 2014), the association between maternal SDP and offspring internalizing behaviors is less clear (Tiesler and Heinrich 2014; Menezes et al. 2013; Ashford et al. 2008; Ekblad et al. 2010; Indredavik et al. 2007; Lavigne et al. 2011; Monshouwer et al. 2011; Orlebeke et al. 1999; Rückinger et al. 2009; Brion et al. 2010; Moylan et al. 2015) and may require large datasets to detect small effects.

The association between maternal SDP and offspring externalizing and internalizing behaviors may be due to a causal effect of maternal SDP on offspring behavior, or to common genetic or environmental effects, and these explanations, clearly, are not mutually exclusive. In the latter case, the transmission from mother to child of genes with pleiotropic effects may result in an association between maternal SDP and offspring externalizing and internalizing behavior. Compared to non-smoking women, women who smoke during pregnancy have lower education and socioeconomic status, are younger, are more often single, and display more psychopathology, both in adulthood and in youth (Knopik 2009; Rogers 2009; Roza et al. 2008; Tong 2009; Kodl and Wakschlag 2004). In addition, mothers who smoked during pregnancy tend to have a partner who smoked as well (Boomsma et al. 1994; Homish et al. 2012). Paternal SDP has been related to lower educational attainment and hazardous drinking (Everett et al. 2007). Pleiotropic effects underlying the association between parent smoking and these variables in adults result in parents who smoke having a higher change of offspring with behavioral problems.

Various approaches have been taken to investigate the causal relationship between SDP and offspring behavior. First, comparing effects of maternal and paternal SDP on offspring problems can help to determine whether the association is causal. Specifically, causal effects originating in the intrauterine environment are consistent with a stronger relationship of prenatal maternal (than paternal) smoking with offspring psychopathology. Adopting this approach, Roza et al. (2008) and Langley et al. (2012) found no support for a different effect of maternal and paternal SDP. However, Brion et al. (2010) and Nomura et al. (2010) obtained results consistent with causal effects of SDP on offspring externalizing behaviors at ages 3–4. Keyes et al. (2014) studied SDP in an historical US sample, ascertained when SDP was common (early 60s). They observed a significant relationship between maternal and paternal SDP and offspring hyperactivity at age 10. The inclusion of covariates rendered the effect of paternal SDP statistically insignificant, which is consistent with a causal interpretation.

A second approach to studying causality is by statistically correcting confounding influences. In a large population-based cohort of Dutch adolescents (at about age 11 years), Monshouwer et al. (2011) included as covariates maternal age at birth, maternal alcohol use during pregnancy, maternal or paternal daily smoking, maternal or paternal history of internalizing and externalizing problems, family socioeconomic status, problems during pregnancy or childbirth, and birth weight. Given this adjustment, associations between maternal SDP and adolescent externalizing problems and substance use disappeared, suggesting that the association was due to confounding influences, not to causal effects of prenatal smoking. Lavigne et al. (2011) and Roza et al. (2008) reported similar results. In contrast, Ekblad et al. (2010) observed that prenatal maternal smoking remained significantly associated with offspring (age 0–20 years) externalizing problems and internalizing behaviors, after adjusting for maternal age, parity, and psychiatric morbidity (established before birth of the child), and the child’s sex, gestational age, birth weight, and Apgar score. For similar results, see Boutwell et al. (2011), Cornelius et al. (2011), and Paradis et al. (2011). Correcting for paternal smoking, and maternal education, age, alcohol consumption and internalizing symptoms, Moylan et al. (2015) observed a dose–response relationship between the amount smoked during pregnancy and the severity of internalizing problems (anxiety and depression) in offspring from 1.5 to 5 years.

A third approach to establishing causality is by means of within-family designs, in which the association can be examined while taking into account genetic and environmental effects common to parental SDP and offspring psychopathology. Silberg et al. (2003), using structural equation modeling in a sample of twin boys (12–17 years) and their mothers, concluded that the familial transmission of risk factors for conduct disorder, rather than the causal effects of SDP, explained the association between maternal prenatal smoking and boys’ conduct disorder. D’Onofrio et al. (2008) studied externalizing behavior in a sample of children of twins, siblings, and cousins (4–10 years). The comparison of offspring who had been exposed to maternal SDP with their non-exposed siblings, revealed a significant, but weak, association with externalizing problems consistent with a causal effect. Kuja-Halkola et al. (2014) compared siblings discordant for maternal SDP, and found that pregnancy outcomes (e.g., birth weight) were consistent with the causal model, but long term cognitive and externalizing outcomes were not.

Thapar et al. (2009) studied ADHD in offspring (4–11 years) of mothers, who had become pregnant through assisted reproductive technologies. The mothers were either genetically related or unrelated to their offspring (some mothers were surrogate mothers, others the recipient of donated oocytes or embryos). The association between maternal SDP and offspring ADHD was only observed in genetically related mother–offspring pairs, implicating common (pleiotropic) genetic factors. In contrast, Gaysina et al. (2013) looked at conduct disorders in offspring (4–10 years) of mothers to whom they were genetically related or unrelated. Their results suggested a causal effect of smoking, as they observed an effect of maternal SDP in both groups of offspring. Ellingson et al. (2014) studied childhood cognitive functioning, temperament, and externalizing longitudinally in siblings (5–14 years). In a multilevel model, they distinguished between within-family and between-family effects of maternal SDP. Controlling for the between-family covariates, rendered within family relationship between SDP and externalizing insignificant, thus casting doubt on the causal hypothesis. Skoglund et al. (2014) studied SDP and ADHD in a Swedish sample, including cousins and siblings of the offspring. The inclusion of between and within family covariates rendered the relationship between maternal SDP and ADHD statistically insignificant, which is inconsistent with the causal hypothesis.

In summary, the results concerning the causal role of maternal SDP in offspring externalizing are mixed, and the results concerning internalizing are too few to arrive at a sensible assessment of the role of SDP (Tiesler and Heinrich 2014). The aim of the present paper is to present two tests of the causal effects of maternal SDP on offspring internalizing and externalizing behaviors in a large population-based sample of 3-year-old children in the Netherlands Twin Register (NTR). Like others (Keyes et al. 2014; Langley et al. 2012; Nomura et al. 2010), we examined the possible direct causal effect of maternal smoking on dimensions of externalizing and internalizing by comparing the associations of maternal and paternal prenatal smoking with dimensions of offspring externalizing and internalizing in the offspring. A stronger effect of maternal SDP is consistent with a causal effect.

Second, we compared the offspring of mothers who continued to smoke during pregnancy to offspring of mothers who quit smoking before they became pregnant (Piper et al. 2012; Robinson et al. 2010). By limiting the analyses to mothers who all had smoked in the year prior to conception we attempted to control for differences between smoking and non-smoking mothers in genetic risk for smoking and comorbid externalizing problems. Under the strong assumption that mothers who quit are comparable to mothers who do not, this may provide additional support for the causal effect of maternal SDP.

## Methods

### Sample

The Netherlands Twin Register (NTR) was established around 1987 at the VU University in Amsterdam, the Netherlands (Boomsma et al. 2006). At the NTR, twins are recruited after birth, and followed longitudinally. At age 3, parental reports on externalizing problems and internalizing psychopathology, health, school performance, and socioeconomic status are collected. We refer to Bartels et al. (2007b) and Van Beijsterveldt et al. (2013) for details on data collection and participation rates. In birth cohorts 1986–2003, the attrition rate between the survey collected before age one (survey 1) and at age three (survey 3) was 32.7 %. A non-response analysis showed that in the families that dropped out, more mothers and fathers smoked during pregnancy (4.9 and 4.4 % difference, respectively), more mothers and fathers were born outside the Netherlands (about 4.0 % difference), and the children were on average about 32 g lighter at birth. We note that 39 % of the dropouts were not permanent, as they participated in later surveys, when their children were 5, 7, 10, or 12 years old. Maternal reports collected at age three were available in 15,228 twin pairs. About 95 % of the parents were born in the Netherlands, about 2.5 % in a western country other than the Netherlands, and about 2.5 % in a non-western country. Over 99 % of the children were born in the Netherlands.

### Measures

Externalizing and internalizing behaviors at age three were assessed by means of maternal reports based on the Dutch version of the Child Behavior Checklist/2–3 (CBCL/2–3; Achenbach and Rescorla 2001; Verhulst et al. 1997). Externalizing was assessed with the oppositional, aggression, and overactive subscales. The sum of all items in these scales forms the broadband scale externalizing problems (denoted “externalizing”). Internalizing was assessed with the withdrawn and anxious/depressed subscales. The sum of the items in these scales forms the broadband scale internalizing problems (denoted “internalizing”). Of the 15,228 pairs, the data of 14,870 pairs were complete for both twins (97.6 %). Socio-economic status (SES) was scored according to the Standard Classification of Occupations (Statistics Netherlands 2001). If this information was not available (3.7 %), SES was scored according to the Erikson–Goldthorpe–Portocarero occupational classes combined with parental level of education (Erikson et al. 1979). SES was coded using a three point scale (low, middle and high SES).

Maternal reports on parental smoking during the pregnancy were obtained on average 8.4 months after the twins were born. Mothers were asked whether they or the father had smoked during the pregnancy, and, if so, how much they had smoked, i.e., less or more than ten cigarettes a day. In the group of mothers who smoked during pregnancy (N = 3238) data were available on the trimester of the pregnancy, in which the mother and father had smoked. In an early version of the survey, the answer categories on this question were ‘irregularly’, or ‘throughout the entire pregnancy’. In later versions of the survey, mothers were more specifically asked about smoking in the first and last trimester of the pregnancy (N = 11,023: not smoking; N = 391: month 1–3; N = 227: month 6–9; N = 2191: month 1–9). Finally, mothers were asked if they had consumed alcohol during the pregnancy. Data on whether the mother had ever smoked, maternal age at birth, offspring sex, alcohol consumption, and birth weight were obtained from the same surveys.

### Analyses

The analyses were carried out in SPSS 21 (IBM Corp. Released 2012) and in OpenMx (Boker et al. 2011). We first calculated the twin correlations for MZ and DZ pairs, and fitted ACE or ADE models depending on the phenotypic correlations using OpenMx. In these analyses we included sex as a covariate. We used OpenMx specifically to obtained confidence intervals of the standardized variance components (e.g., h^2^). To test the causal hypotheses, we carried out regression analyses in linear mixed models using the SPSS linear mixed procedure. In so doing, we regressed the phenotypic scores of the twins on the predictors of main interest and several covariates. We simultaneously fitted the ACE or ADE model to the residuals to account for the residual twin covariance (McArdle and Prescott 2005; Rabe-Hesketh et al. 2008). The choice of ACE or ADE was based on the results of the prior OpenMx analyses. All analyses were carried out using raw data maximum likelihood estimation.

**Fig. 1:**
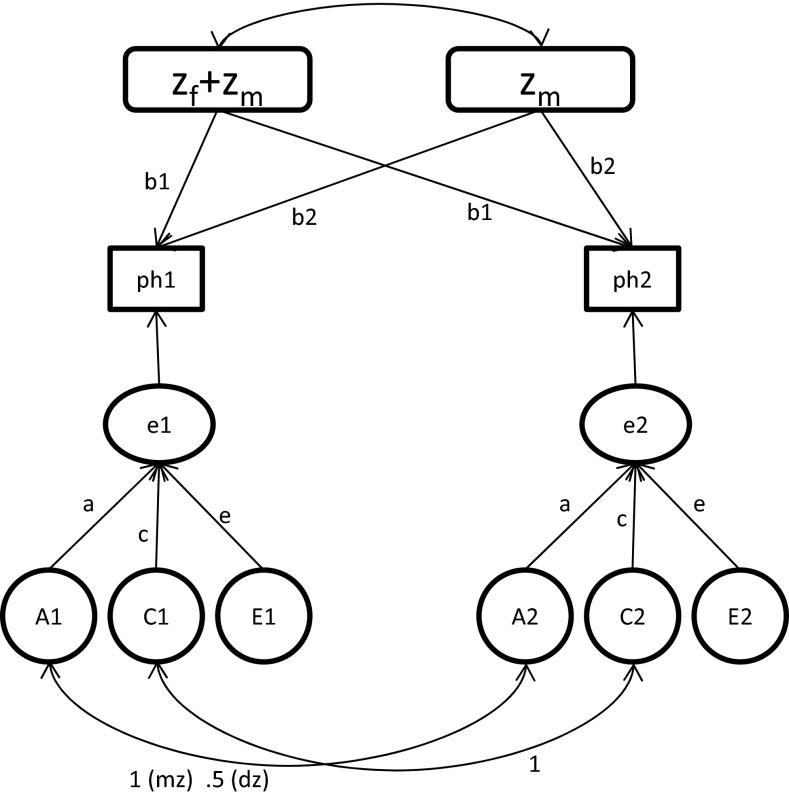
Path diagram of the model. Ph1 (ph2) is the phenotype as observed in twin 1 (twin 2). The variables z_f_ and z_m_ are the standardized smoking variables in father and mother, respectively. The residuals (e1, e2) are subject to a ACE decomposition, to account for the residual covariance (in the analysis of overactiveness and anxious depression, we fitted an ADE model). As explained in the text, the parameter b_2_ of main interest, as b_2_ > 0 implies that maternal SDP has a greater influence than paternal SDP, i.e., consistent with a direct causal effect of SDP on the phenotype

To test the difference between the contributions of paternal and maternal smoking to variance in the CBCL test scores, we standardized the paternal and maternal smoking variables (denoted z_f_ and z_m_), so that their variances were equal to one. We added these paternal and maternal smoking z-scores to create a parental sum z-score (z_f_ + z_m_). We then included the sum score and the maternal smoking z-score as predictors, along with the covariates sex, SES, birth weight, alcohol consumption of the mother during pregnancy, and age of the mother at birth. Limiting ourselves to the smoking variables for convenience, the test is based on the following. Given y = b_0_ + g_1_ * z_f_ + g_2_ * z_m_ + e (discarding subject subscripts), we want to test whether the contribution to the explained variance of z_m_ (g_1_^2^ * var(z_f_) = g_1_^2^) equals that of z_f_ (g_2_^2^ * var(z_m_) = g_2_^2^), in the total decomposition of variance (i.e., g_1_^2^ + g_2_^2^ + 2 * g_1_ * g_2_ * r(z_m_, z_f_)). To this end, we may fit y = b_0_ + b_1_ * z_f_ + (b_1_ + b_2_) * z_m_ + e, so that the null hypothesis of interest is b_2_ = 0 (vs. b_2_ > 0). This can be done conveniently by fitting y = b_0_ + b_1_ * (z_f_ + z_m_) + b_2_ * z_m_ + e, and testing the estimate of b_2_. An estimate of b_2_ significantly greater than zero is consistent with the causal model, as it implies that maternal SDP has a greater effect, in terms of explained variance, than paternal SDP. As mentioned above, the residual (e) was subject to a ACE or ADE decomposition to account for the dependency of the twins (conditional on the predictors z_m_, z_f_ + z_m_, and other covariates). The model is depicted Fig. 1.

The second analyses were performed in the subsample of mothers who had smoked in the year prior to conception. We created four groups of these mothers: (1) mothers, who did not smoke during pregnancy (i.e., quit smoking; N = 1300); (2) mothers, who smoked in months 1–3 (first trimester; N = 294); (3) those who smoked in months 6–9 (3rd trimester; N = 146); and (4) those who smoked in throughout pregnancy (N = 1388). In the second analyses, we compared the ratings of the mothers who quit smoking (coded 0) to the mothers who smoked in trimester 1, trimester 3, or throughout (groups 2 to 4 combined, coded 1). We included this group variable and the covariates sex, SES, birth weight, alcohol consumption of the mother during pregnancy, and age of the mother at birth. The analyses were conducted in SPSS linear mixed by regressing the phenotypic scores of the twins on the group variable and the covariates. As in the first analyses, we accommodated the residual twin covariance (i.e., conditional on the predictors) by fitting an ACE or ADE model.

## Results

### Sample characteristics

The prevalence of maternal SDP was 19.5 %, in line with the prevalence reported in the general Dutch population (Lanting et al. 2007; Roza et al. 2008). The distribution of the smoking variable in mothers is N = 10,625 (no smoking), N = 2067 (<10 cigarettes a day), and N = 524 (>10 cigarettes a day). In the fathers, this distribution N = 9670 (no smoking), N = 1945 (<10 cigarettes a day), and N = 972 (>10 cigarettes a day). The parental correlation (Spearman’s ρ) for smoking was 0.376 (*p* < 0.001). The distribution of alcohol consumption in the mothers is N = 12,399 (no alcohol); N = 2319 (<1 glass a week), and N = 427 (>1 glass a week). SES was distributed as follows: N = 3471 (low SES), N = 6973 (middle SES); N = 4731 (high SES). As shown in Table 1, there are no differences between smoking and non-smoking mothers in the distribution of the sex of the offspring. However, there is a strong association between maternal and paternal smoking, between maternal smoking and SES, birth weight (Cohen’s *d* = ~0.27), and between smoking and drinking (all *p* values < 0.001). In addition, mothers who smoked were younger at the time of the birth of their twins (Cohen’s *d* = 0.15; *p* < 0.001).

**Table 1:** Characteristics of mothers who smoked and who did not smoke during pregnancy

	Non-smoking mothers	Smoking mothers	Test of association
	11,948	3238	
N girls 1st born	5978	1518	χ^2^ (1) = 2.63; ns
N boys 1st born	5970	1670	
N girls 2nd born	6021	1642	χ^2^ (1) = 0.10; ns
N boys 2nd born	5927	1596	
Paternal prenatal smoking yes	2660	1911	χ^2^ (1) = 1742*
Paternal prenatal smoking no	8575	1087	
SES low	2301	1154	χ^2^ (2) = 520*
SES medium	5484	1475	
SES high	4130	590	
Mean birth weight (sd) 1st	2560 (543)	2417 (540)	F(1,14983) = 174*
Mean birth weight (sd) 2nd	2505 (552)	2356 (551)	F(1,14975) = 182*
Mean maternal age at birth (SD)	31.2 (3.8)	30.6 (4.0)	F(1,15110) = 674*
Alcohol NO	9844	2536	χ^2^ (2) = 65.3*
Alcohol < 1 glass a week	1789	537	
Alcohol > 1 glass a week	272	154	

* *p* < 0.0001; the total sample size (number of families) is 15,228. Note due to missing values the total sample size, as derived from these counts, is less than 15,228. Standard deviations are given in parentheses (birthweight and maternal age)

Table 2 contains the averages and the standard deviation of the phenotypes observed in the 3-year old twins for 1st and 2nd born twins and the phenotypic relationship with maternal SDP. The effect of smoking is significant (all *p* < 0.001). Judging by the effect sizes (Cohen’s *d*), smoking has the largest effect on externalizing (*d* = ~0.28) and the smallest effect on anxious depression (*d* = ~0.07).

**Table 2:** Means and standard deviations of twins with mothers, who smoked or did not smoke during pregnancy (SDP)

	SDP	N	Mean	SD	*d* ^a^
1st born					
Oppositional	No	11,842	10.10	6.39	0.232
	Yes	3200	11.60	6.70	
Withdrawn	No	11,836	1.13	1.49	0.180
	Yes	3197	1.41	1.78	
Aggression	No	11,860	3.19	2.71	0.270
	Yes	3217	3.94	3.05	
Anxious depression	No	11,816	3.59	3.07	0.071
	Yes	3194	3.81	3.14	
Overactive	No	11,883	2.63	2.13	0.231
	Yes	3217	3.13	2.30	
Internalizing	No	11,811	4.72	4.00	0.123
	Yes	3192	5.22	4.26	
Externalizing	No	11,781	15.93	9.72	0.280
	Yes	3195	18.70	10.48	
2nd born					
Oppositional	No	11,817	9.67	6.41	0.239
	Yes	3194	11.22	6.75	
Withdrawn	No	11,782	1.10	1.49	0.174
	Yes	3189	1.37	1.78	
Aggression	No	11,824	3.02	2.64	0.256
	Yes	3206	3.74	2.98	
Anxious depression	No	11,763	3.40	3.06	0.071
	Yes	3179	3.62	3.16	
Overactive	No	11,848	2.52	2.12	0.227
	Yes	3210	3.01	2.30	
Internalizing	No	11,751	4.51	3.97	0.119
	Yes	3176	4.99	4.26	
Externalizing	No	11,724	15.24	9.70	0.281
	Yes	3177	18.01	10.50	

^a^Differences in means all significant (*p* < 0.001). Effect size is Cohen’s *d* (standard deviation based the pooled estimate)

### Associations between maternal and paternal smoking during pregnancy with externalizing problems and internalizing psychopathology

To determine background covariance structure, we fitted an ACE or ADE model (with sex as the only covariate) depending on the observed MZ and DZ twin correlations. We used OpenMx (Boker et al. 2011) to fit these models and to obtain the 95 % confidence intervals (CI95) of the estimated standardized variance components (these CI95s are shown in brackets). We fitted the ADE model to anxious depression (rMZ = 0.72, rDZ = 0.34; h^2^ = 0.59 [CI95 0.517–0.658], d^2^ = 0.11 [CI95 0.045–0.190], e^2^ = 0.29 [CI95 0.281–0.306]) and the overactive scores (rMZ = 0.69, rDZ = 0.17; h^2^ = 0.0 [0–0.037], d^2^ = 0.70 [0.663–0.715], e^2^ = 0.30 [0.284–0.309]). We fitted the ACE model to the internalizing scores (rMZ = 0.74, rDZ = 0.41; h^2^ = 0.69 [0.649–0.723], c^2^ = 0.07 [0.024–0.091], e^2^ = 0.25 [0.244–0.265]), the withdrawn scores (rMZ = 0.69, rDZ = 0.42; h^2^ = 0.65 [0.615–0.690], c^2^ = 0.07 [0.038–0.104], e^2^ = 0.27 [0.263–0.287]), the externalizing scores (rMZ = 0.83; rDZ = 0.53; h^2^ = 0.56 [0.538–0.595], c^2^ = 0.26 [0.238–0.292], e^2^ = 0.17 [0.160–0.174]), the oppositional scores (rMZ = 0.78, rDZ = 0.49; h^2^ = 0.58 [0.458–0.612], c^2^ = 0.20 [0.176–0.235], e^2^ = 0.21 [0.204–0.222]) and the aggression scores (rMZ = 0.83, rDZ = 0.42; h^2^ = 0.71 [0.679–0.743], c^2^ = 0.12 [0.088–0.149], e^2^ = 0.17 [0.162–0.177]). We observed significant sex effects with boys scoring higher on internalizing (*p* < 0.001), externalizing (*p* < 0.001), oppositional (*p* = 0.01), withdrawn (*p* < 0.001), aggression (*p* < 0.001), overactive behavior (*p* < 0.001), and anxious depression (*p* = 0.001). These results are consistent with previous analyses of these data (Derks et al. 2004; Hudziak et al. 2003; Bartels et al. 2004).

The results of the test of the first causal hypothesis concerning the parameter b_2_ (see above) are shown in Table 3. The focus is on the significance of the regression coefficient associated with maternal smoking (denoted b_2_ above). In our formulation of the regression model, as explained above, this parameter represents the difference in variance explained by paternal and maternal SDP. As we conducted seven tests, we adopted an α of 0.05/7 = ~0.007. As we expect the effect of maternal SDP to be greater than paternal SDP, we adopt a one-sided test. By this criterion, we find that the parameter of interest is significantly greater than zero in the analyses of externalizing, aggression, oppositional, and withdrawn. Expressing the effect sizes in terms of the R^2^ change observed by adding maternal smoking to the rest of the predictors, we find that the effect sizes are small. The R^2^ change ranges from 0.012 % (i.e., 1.2/1000th of 1 %; anxious depression) to 0.083 % (8.3/1000th of 1 %; externalizing). We do not consider statistical tests of the other predictors, as they are not of interest here.

**Table 3:** *P* values in the regression of the dependent phenotype (column 1) on the covariates (columns 2–6) and the predictor of interest (“maternal vs. paternal SDP”; column 11)

Dependent phenotype	SES	SEX	Birth weight	Alcohol	Age mother	Mean parental SDP	Maternal vs. paternal SDP^a^	R^2^ total (%)	R^2^ change (%)	b_2_ maternal vs. paternal SDP
Internalizing	<0.001	<0.001	<0.001	0.23	<0.001	0.783	0.011	1.7	0.029	0.132 (0.058)
Anxious depression	<0.001	<0.001	0.110	0.006	<0.001	0.957	0.060	1.0	0.012	0.068 (0.043)
Withdrawn	<0.001	<0.001	<0.001	0.022	<0.001	0.426	*0.002*	2.4	*0.048*	*0.064 (0.022)*
Externalizing	<0.001	<0.001	<0.001	0.421	<0.001	0.001	<*0.001*	4.9	*0.083*	*0.520 (0.146)*
Overactiveness	<0.001	<0.001	<0.001	0.134	<0.001	0.001	0.009	4.9	0.028	0.067 (0.028)
Aggression	<0.001	<0.001	<0.001	0.038	<0.001	<0.001	*0.002*	8.2	*0.061*	*0.117 (0.039)*
Oppositional	<0.001	0.002	0.009	0.341	<0.001	0.020	<*0.001*	2.8	*0.080*	*0.345 (0.095)*

The *p* values of interest are given in column “maternal vs. paternal SDP” (column 8). The *p* values smaller than the alpha (0.05/7 = 0.007) are italicized. The columns “R^2^ total” (9) and “R^2^ change” (10) contain the total R^2^ (explained variance) and the R^2^ change due to the addition of maternal SDP. Column “b_2_ maternal vs. paternal SDP” (11) contains the estimated difference (standard error in parentheses) of the regression coefficient (b_2_) between father and mother

^a^One-sided test of difference of effect of paternal and maternal SDP

The results of the second analyses are shown in Table 4. In these analyses we tested the difference in the phenotypic scores of the offspring of mothers, who quit smoking prior to conception (N = 1300), and mothers, who continued to smoke. Note that all mothers had smoked in the year prior to conception and were well established smokers. On average the mothers who quit prior to conception had smoked for a period of 10.7 years. Mothers who continued to smoke during pregnancy had smoked for 12.3 (months 1–3), 11.8 (months 6–9), or 13.3 years (months 1–9). We again adopted an α of ~0.007 (0.05/7), and focused on the main effect of smoking. The results in Table 4 indicate clearly that there is an effect on externalizing, overactive behavior, aggression, and oppositional behavior. We see no effect on the internalizing scores (internalizing, anxious depression, withdrawn behavior). The effect sizes (R^2^ change) range from 0.1 (1/100th of 1 %; anxious depression) to 0.52 (5.2/100th of 1 %; externalizing). We explored the differences in phenotypic scores of twins, whose mothers quit and mothers who continued to smoke during the first trimester, the 3rd trimester, or throughout pregnancy. Table 5 contains the parameter estimates which represent the mean differences relative to the condition no SDP (i.e., mothers who quit). The results suggest that smoking during the first trimester has no detectable effect, given the present sample size.

**Table 4:** *P* values in the regression of the dependent phenotype (column 1) on the covariates and the predictor of interest (“maternal SDP”; column 7)

Dependent phenotype	SES	SEX	Birth weight	Alcohol	Age mother	Maternal SDP	R^2^ total (%)	R^2^ change (%)
Internalizing	<0.001	<0.001	<0.001	0.247	<0.001	0.071	2.5	0.15
Anxious depression	<0.001	0.001	0.012	0.147	0.001	0.193	1.4	0.10
Withdrawn	<0.001	<0.001	<0.001	0.876	<0.001	0.068	3.0	0.14
Externalizing	<0.001	<0.001	<0.001	0.289	<0.001	<*0.001*	4.0	*0.52*
Overactiveness	<0.001	<0.001	<0.001	0.048	<0.001	*0.004*	4.5	*0.28*
Aggression	0.001	<0.001	0.004	0.730	<0.001	*0.001*	7.5	*0.42*
Oppositional	<0.001	0.004	0.001	0.525	<0.001	<*0.001*	2.9	*0.43*

The *p* values of interest are associated with maternal SDP (column 7). These *p* values concern the omnibus test of an effect of maternal SDP (see Table 5 for contrasts relative to no SDP). The *p* values smaller than the alpha (0.05/7 = 0.007) are italicized. The columns “R^2^ total” (8) and “R^2^ change” (9) contain the total R^2^ (explained variance) and the R^2^ change due adding SDP

**Table 5:** The differences in phenotypic scores of twins of mother who quit smoking (reference group) compared to mothers who smoked in the first trimester (“SDP months 1–3”), the last trimester (“SDP months 6–9”), or throughout (“SDP months 1–9”)

Dependent phenotype	SDP months 1–3	SDP months 6–9	SDP months 1–9
Internalizing	0.247 (0.234)	0.764 (0.317)*	0.222 (0.143)
Anxious depression	0.142 (0.169)	0.458 (0.229)*	0.130 (0.104)
Withdrawn	0.120 (0.096)	0.309 (0.130)*	0.092 (0.058)
Externalizing	0.762 (0.578)	2.21 (0.783)**	1.48 (0.354)**
Overactiveness	0.224 (0.115)	0.397 (0.156)*	0.208 (0.070)**
Aggression	0.147 (0.161)	0.291 (0.219)	0.400 (0.099)**
Oppositional	0.384 (0.371)	1.52 (0.504)**	0.854 (0.227)**

Two sided tests: * *p* < 0.05; ** *p* < 0.005

## Discussion

In a large sample of Dutch families, we obtained some support for a direct causal effect of maternal prenatal tobacco exposure on externalizing dimensions of behavior in offspring at age 3 years. Associations of maternal prenatal tobacco exposure with offspring internalizing dimensions at age three were largely absent, with the possible exception of the withdrawn dimension, see Table 4. The observed effects are small in terms of R^2^, but nevertheless add provisional support for causal effects of maternal prenatal tobacco exposure on externalizing behaviors in 3 year olds (Agrawal et al. 2010; D’Onofrio et al. 2008; Ekblad et al. 2010; Knopik 2009).

Weaker associations between prenatal smoking and offspring internalizing behaviors than with externalizing behaviors have previously been observed, but why these associations are less strong, is unclear (Lavigne et al. 2011; Monshouwer et al. 2011; Orlebeke et al. 1999). Underlying genetic factors may in part explain this difference. There is stronger evidence for genetic pleiotropy of substance use and externalizing problems than there is for substance use and internalizing psychopathology (Edwards et al. 2011; Hicks et al. 2011; Kendler et al. 2003; Stephens et al. 2012) and thus even without any causal effects of prenatal smoking, an association of maternal smoking and externalizing offspring behavior is expected as mothers pass on their risk genes to their offspring. The association of prenatal smoking with offspring externalizing problems may be further amplified by interactions between offspring genes and prenatal tobacco exposure. Prenatal tobacco exposure interacts with fetal MAOA genotype and with several dopaminergic genes, leading to increased offspring externalizing problems in children who were already genetically susceptible (Brennan et al. 2011; Kahn et al. 2003; Langley et al. 2008; Neuman et al. 2007; Wakschlag et al. 2009). There is less evidence for such genotype × prenatal tobacco exposure effects for internalizing, although Hsieh et al. (2010) observed an interaction between maternal prenatal passive smoking and a fetal metabolic gene (CYP1A1), which resulted in more offspring internalizing at age two. Cents et al. (2012) examined effects of 5-HTTLPR genotype and prenatal tobacco exposure on offspring internalizing at age three. Carrying a short allele of the 5-HTTLPR polymorphism in combination with prenatal tobacco exposure, predicted increased internalizing psychopathology at age three. However these results did not replicate (Geels et al. 2012).

Other mechanisms may also play a role. Maternal SDP is related to maternal depression, which in turn predicts offspring *aggression* (Brook et al. 2006; Lancaster et al. 2010), and this ties in with observations that young children with depression may express problems partly through indirect, ‘masked’ symptoms, like aggression and somatic complaints (Luby et al. 2003). Knopik et al. (2012) reviewed mechanisms of DNA methylation patterns and altered miRNA expression associated with maternal cigarette SDP, suggesting and outlining biological pathways that can be affected by prenatal maternal smoking.

We note some limitations of this study, including the absence of information on maternal psychopathology. The association between prenatal maternal smoking may disappear after maternal psychopathology is included (Lavigne et al. 2011; Monshouwer et al. 2011; Roza et al. 2008), however in other studies, the association was attenuated, but remained significant (Boutwell et al. 2011; Cornelius et al. 2011; Ekblad et al. 2010; Paradis et al. 2011). However, our conclusions rest in part on the comparison between maternal and paternal SDP, which showed that maternal SDP was more strongly associated with offspring externalizing problems. This conclusion probably is robust given the effects of parental psychopathology, since maternal smoking often co-occurs with paternal SDP, and both are related to adverse circumstances (Everett et al. 2007; Rogers 2009; Roza et al. 2008; Tong 2009).

We did not include information on post-natal parental smoking. Children, whose mothers smoked during pregnancy, are more likely to also be exposed to second-hand smoke in childhood (Knopik 2009). Environmental tobacco exposure has been linked to increased risk of hyperactive/inattention and externalizing problems (Kabir et al. 2011; Tiesler et al. 2011). Including this information enables separating effects of prenatal tobacco exposure from passive smoking during childhood (Schlotz and Phillips 2009; Thapar and Rutter 2009).

Furthermore, using maternal reports on maternal and paternal smoking, as well as on offspring externalizing and internalizing problems, could introduce projection bias (Bartels et al. 2007a). Additional analyses of paternal ratings of offspring behavior (available for a subsample of 6598–6631 children) yielded the same pattern of results. Retrospective self-reports on SDP may underestimate prenatal tobacco exposure, but a study comparing retrospective self-reports on prenatal smoking to prospective measurements and cotinine assessments, showed that generally, all types of measurements performed equally well (Pickett et al. 2009). In addition, reports of smoking among relatives are very highly correlated with those relatives’ self-reports (Kendler et al. 2002). Moreover, information on parental SDP was gathered on average 8.4 months after birth of the twins, minimizing recall bias effects. Finally in our comparison of offspring of mothers, who quit smoking before pregnancy, and mothers, who continued to smoke, we assumed that these groups were comparable with respect to environmental and genetic background variables. This may not be the case, however, as the ability to quit, even among established smokers may be related to genetic influences (Freathy et al. 2009).

In summary, the results concerning the associations between maternal SDP and offspring externalizing behavior at age three are consistent with a small causal (direct) effect of maternal SDP. The results concerning the associations between maternal SDP and offspring internalizing behavior involve no causal (direct) effect of maternal smoking, or perhaps an effect that is too small to be detected with the present sample size.
